# Core binding factor subunit β plays diverse and essential roles in the male germline

**DOI:** 10.3389/fcell.2023.1284184

**Published:** 2023-11-02

**Authors:** Mustika Rahmawati, Kassie M. Stadler, Blanca Lopez-Biladeau, Tia M. Hoisington, Nathan C. Law

**Affiliations:** ^1^ Department of Animal Sciences, College of Agricultural, Human, and Natural Resources Sciences, Washington State University, Pullman, WA, United States; ^2^ School of Molecular Biosciences, College of Veterinary Medicine, Washington State University, Pullman, WA, United States

**Keywords:** spermatogonial stem cell (SSC), spermatogenesis, RUNX family, core-binding factor beta (CBFβ), fate, germline stem cell, germline development

## Abstract

Much of the foundation for lifelong spermatogenesis is established prior to puberty, and disruptions during this developmental window negatively impact fertility long into adulthood. However, the factors that coordinate prepubertal germline development are incompletely understood. Here, we report that core-binding factor subunit-β (CBFβ) plays critical roles in prepubertal development and the onset of spermatogenesis. Using a mouse conditional knockout (cKO) approach, inactivation of *Cbfb* in the male germline resulted in rapid degeneration of the germline during the onset of spermatogenesis, impaired overall sperm production, and adult infertility. Utilizing a different Cre driver to generate another *Cbfb* cKO model, we determined that the function of CBFβ in the male germline is likely limited to undifferentiated spermatogonia despite expression in other germ cell types. Within undifferentiated spermatogonia, CBFβ regulates proliferation, survival, and overall maintenance of the undifferentiated spermatogonia population. Paradoxically, we discovered that CBFβ also distally regulates meiotic progression and spermatid formation but only with *Cbfb* cKO within undifferentiated spermatogonia. Spatial transcriptomics revealed that CBFβ modulates cell cycle checkpoint control genes associated with both proliferation and meiosis. Taken together, our findings demonstrate that core programs established within the prepubertal undifferentiated spermatogonia population are necessary for both germline maintenance and sperm production.

## Introduction

Prior to puberty, the male germline transitions from an immature state to a productive state capable of supporting sperm production through the process known as spermatogenesis. During this time, the foundation for lifelong spermatogenesis is established. First, the undifferentiated spermatogonia population is formed, which includes spermatogonial stem cells (SSC), that will maintain the germline indefinitely by producing daughter cells that eventually differentiate to form sperm. Second, during prepubertal development, the first spermatocytes are produced, which become the replenishing source of retinoic acid (RA) necessary to sustain spermatogenesis (Raverdeau et al., 2012; Tong et al., 2013; Teletin et al., 2018; Griswold, 2022; Topping and Griswold, 2022). Finally, more recent studies have demonstrated that lumicrine factors such as NELL2 and NICOL are produced during the onset of spermatogenesis to promote the maturation of the epididymis, which is necessary for fertility (Kiyozumi et al., 2020; Kiyozumi et al., 2023). Additionally, accumulating studies collectively indicate that prepubertal germline development, including during the onset of spermatogenesis, is a sensitive window to disruptions. Epidemiological studies in humans and toxicology studies in rodents indicate that prepubertal and neonatal exposure to estrogenic compounds such as bisphenol A, bisphenol S, and ethinyl estradiol or various chemotherapeutics have long-term, negative impacts on spermatogenesis into adulthood (Muller et al., 1988; Cabral et al., 2014; Vrooman et al., 2015; Chow et al., 2016; Horan et al., 2017; Okada et al., 2020). Thus, prepubertal exposure to such gonadotoxic compounds is thought to underlie forms of male subfertility or infertility with developmental origins (Allen et al., 2018). However, the factors that regulate the onset of spermatogenesis and are susceptible to disruption remain largely unknown.

The developmental events that comprise postnatal maturation of the male germline leading up to puberty in humans are incompletely characterized, but the process has been well-studied in mice. At birth, mouse germ cells reside within the testis as mitotically inactive precursors, known as prospermatogonia or gonocytes. Prospermatogonia asynchronously re-enter the cell cycle during the first 1–2 days postnatally (P1-P2) (Kluin and Rooij, 1981; McGuinness and Orth, 1992; Nagano et al., 2000; Yoshida et al., 2006; Law et al., 2019; Du et al., 2021). In subsequent developmental days, prospermatogonia transition to form spermatogonia of different functional capacities. Some prospermatogonia form the undifferentiated spermatogonia population, which includes both SSCs that maintain the germline indefinitely and more progenitor-like spermatogonia poised for differentiation (Yoshida et al., 2006; Hermann et al., 2015; de Rooij, 2017; Pui and Saga, 2017; Law et al., 2019). Meanwhile, other prospermatogonia respond to RA and directly transition into terminal differentiation as differentiating spermatogonia (de Rooij, 1998; de Rooij, 2001; Yoshida et al., 2006; Snyd et al., 2010; Snyder et al., 2011; Niedenberger et al., 2015; Agrimson et al., 2016). The first differentiating spermatogonia committed to terminal differentiation form meiotic spermatocytes around P10 that will then undergo two rounds of meiosis to form haploid spermatid beginning at approximately P20 (Bellve et al., 1977; Ernst et al., 2019). Spermatids then undergo a dramatic morphological transformation, known as spermiogenesis, and develop into the first spermatozoa around P28 (Oakberg, 1956; Bellve et al., 1977; Ernst et al., 2019). This initial cohort of germ cells that forms spermatozoa is collectively known as the first round of spermatogenesis (Kluin and Rooij, 1981; Yoshida et al., 2006). Subsequent rounds of spermatogenesis develop asynchronously throughout the testis until all germ cell types are continuously produced and steady-state spermatogenesis is reached (Yoshida et al., 2006; de Rooij, 2017; Hermann et al., 2018; Grive et al., 2019). Concurrent with the first round of spermatogenesis, the undifferentiated spermatogonia population, including nascent SSCs, undergoes rapid proliferative expansion to establish the replenishing reservoir that ultimately supports continuous sperm production (Drumond et al., 2011; Law et al., 2019).

Numerous studies have highlighted the involvement of the Runt-related transcription factor (RUNX) family in key developmental transitions across several cell lineages (Inoue et al., 2008; Ito et al., 2015; Seo and Taniuchi, 2020). The mammalian RUNX family is composed of three DNA-binding proteins (RUNX1, RUNX2, and RUNX3) that each contain a conserved Runt domain that interacts with core-binding factor subunit-β (CBFβ) (Tahirov et al., 2001; Ito et al., 2015). Although it does not directly associate with DNA, CBFβ stabilizes the interaction between RUNX proteins and DNA, and protects RUNX proteins from ubiquitin-mediated degradation (Ogawa et al., 1993; Huang et al., 2001). Furthermore, genetic mutations of *Cbfb* or disruption of the CBFβ-RUNX protein interaction compromises transcriptional regulation by the complex (Ito et al., 2015). Therefore, CBFβ is instrumental for the overall functional activity of the RUNX protein family. Dysregulation of CBFβ in mammals has been implicated in a multitude of diseases, including cancer, skeletal disorders, and immune-related conditions (Look, 1997; Speck et al., 1999; Komori, 2003; Roudaia et al., 2009). Global knockout of *Cbfb* in mice results in embryonic lethality due to fetal hemorrhaging (Okuda et al., 1996; Wang et al., 1996). Therefore, studies of CBFβ often require tissue-specific conditional deletion in mouse models to explore function. Oncology studies suggest that CBFβ acts as a tumor suppressor by inhibiting pro-tumorigenic signaling in breast cancer (Pegg et al., 2019; Malik et al., 2021). Also, studies in osteogenesis indicate that CBFβ regulates osteoblast differentiation, chondrocyte proliferation, and lineage maturation during postnatal cartilage formation (Tian et al., 2014; Qin et al., 2015). Furthermore, studies revealed that CBFβ is necessary to maintain hematopoietic and epithelial stem cells in mice by promoting proliferation and differentiation (Speck et al., 1999; Link et al., 2010; Kurosaka et al., 2011). Collectively, these studies highlight that the CBFβ-RUNX complex modulates the expression of genes essential for proper development across various tissues.

Despite its conserved role in developmental regulation, the function of CBFβ in male germline development and spermatogenesis has not been explored. Therefore, in the present study, we generated *Cbfb* conditional knockout (cKO) mice to assess the functional role of CBFβ in testis development. Using the cKO mice model, we found that the male germline rapidly degenerates during prepubertal development during the onset of spermatogenesis and adult cKO males are sterile. Through the use of different cKO models, we discovered that while CBFβ is expressed in differentiating spermatogonia, spermatocytes, and spermatid, it is functionally dispensable for spermatogenesis within these germ cell types. In contrast, using genetic approaches to conditionally inactivate *Cbfb* in undifferentiated spermatogonia, the germline progressively declines and distal germ cell maturation was disrupted, including spermatocyte meiotic progression, spermatid formation, and overall sperm production. Finally, spatial transcriptomic analyses revealed that *Cbfb* regulates a number of molecular frameworks within different germ cell types, but commonly regulates genes associated with cell cycle checkpoint control. Collectively, our studies not only demonstrate that *Cbfb* is essential for male germline development, but also reveal that components of the terminal differentiation program underlying spermatogenesis are established in undifferentiated spermatogonia.

## Results

### Conditional ablation of *Cbfb* leads to male infertility

**FIGURE 1:**
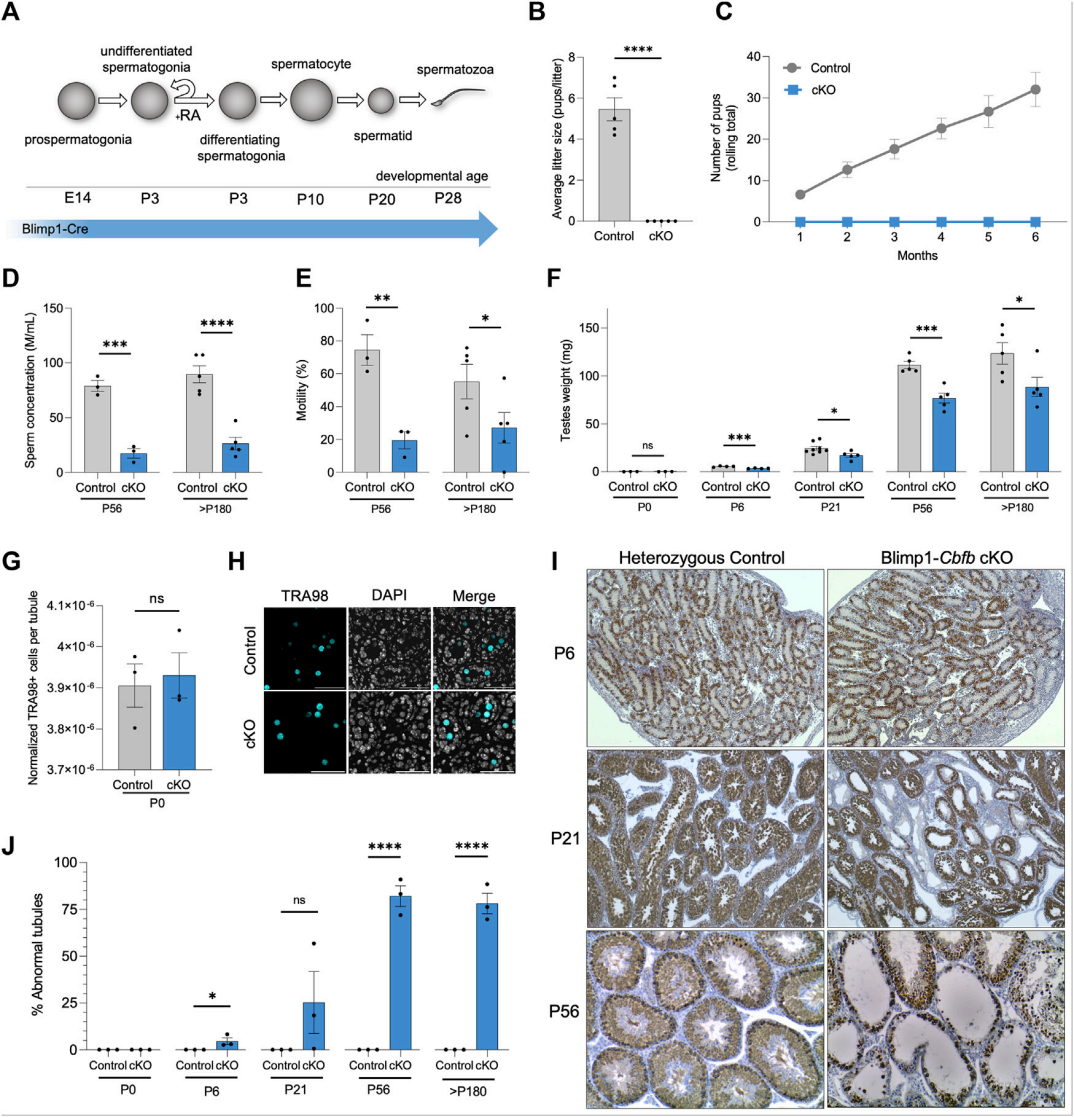
Conditional inactivation of *Cbfb* in the male germline leads defects in prepubertal development and adult infertility. **(A)** Schematic representation of germ cell types that form during postnatal development. Expression of Blimp1-cre is indicated. **(B,C)** Average litter size **(B)** and cumulative number of pups **(C)** from heterozygous control and Blimp1-*Cbfb* cKO males bred to wild-type females throughout the breeding trial. **(D,E)** Sperm concentration **(D)** and percent motile sperm **(E)** at P56 and >P180 were analyzed using CASA. **(F)** Mean paired testis weight throughout postnatal testis development. **(G,H)** Immunofluorescence staining of P0 testis cross-sections for all germ cells marked by TRA98+. Quantifications **(G)** presented as TRA98+ cell counts per tubule cross-section normalized cross-sectional area. Representative images presented in **(H)**. **(I,J)** Immunohistochemical analysis of the pan germ cell marker DDX4 (brown) using testis cross-sections from heterozygous control and Blimp1-*Cbfb* cKO males. Representative images **(I)** and quantification of abnormal tubules **(J)** are presented. Quantifications in B-G and J are presented as means ± SEM for *n* = 3–8 biologically independent animals per genotype and age point. *p < 0.05, **p < 0.01, ***p < 0.001; not significant (ns).

To generate a conditional knockout (cKO) model in which *Cbfb* is inactivated from germline throughout prenatal developmental and postnatal life, we utilized Blimp1-Cre^Tg^ and *Cbfb* ^*fl/fl*^ transgenic mouse lines (denoted Blimp1-*Cbfb* cKO) (Figure 1A). Blimp1-CreTg is expressed within the germline starting at approximately embryonic day (E) 6.5 (Ohinata et al., 2005) and thus, serves as an effective approach to inactive *Cbfb* within the germline throughout prenatal and postnatal development. Using this cKO model, *Cbfb* transcript abundance was reduced by 75.3% in Blimp1-*Cbfb* cKO testes compared to heterozygous controls (Supplementary Figure S1A). Next, we evaluated the fertility of male Blimp1-*Cbfb* cKO and heterozygous controls in a 6-month breeding trial. Outcomes of the breeding trial revealed that Blimp1-*Cbfb* cKO males were completely infertile and produced no offspring despite the observation of copulatory plugs (Figures 1B, C). Computer-assisted sperm analysis (CASA) at sexual maturity [postnatal day (P) 56] and the end of the breeding trial (*p* > 180) revealed significantly reduced sperm concentration (77.85% reduction at P56% and 70.53% reduction at *p* > 180; Figure 1D) and sperm motility (70.41% reduction at P56% and 50.89% reduction at *p* > 180; Figure 1E) in Blimp1-*Cbfb* cKO males compared to controls. Also, mean paired testis weights were significantly reduced in Blimp1-*Cbfb* cKO males (Figure 1F), suggesting impaired overall sperm production.

While Blimp1-Cre activity is restricted to the germline within the testis, Cre activity has been reported in somatic tissues (Driskell et al., 2013). Therefore, we sought to determine if pituitary-derived hormones of the hypothalamic-pituitary-gonadal axis that regulate reproductive processes like spermatogenesis were differentially impacted within Blimp1-*Cbfb* cKO males. Outcomes revealed no significant difference in luteinizing hormone (LH) production but a reduction in follicle-stimulating hormone (FSH; Supplementary Figures S1B, C). Importantly, studies of *Fshb* knockout mice demonstrated that while FSH contributes to quantitatively normal levels of sperm production (∼40% reduction with *Fshb* knockout), *Fshb* knockout mice remain fertile (Kumar et al., 1997). Thus, decreased circulating FSH levels cannot fully explain the infertile phenotype observed by Blimp1-*Cbfb* cKO males.

Considering the reduced testis weights observed as early as P6 in Blimp1-*Cbfb* cKO males, we hypothesized that germline disruptions might be present within the testis. Newborn Blimp1-*Cbfb* cKO testes contained a normal complement of germ cells (Figures 1G, H), suggesting that CBFβ is not necessary for prenatal germline development. Therefore, we performed histological evaluations through postnatal development using testis cross-sections and immunostained for the pan germ cell marker DDX4. Histological analysis revealed tubules lacking germ cells as early as P6 (Figure 1I). Interestingly, P21 Blimp1-*Cbfb* cKO testes contained heterogeneous seminiferous tubule phenotypes, including tubules devoid of germ cells, containing only spermatogonia, or containing advanced germ cells but lacking spermatid. These abnormalities were grouped and quantified as a percentage of “abnormal” versus “normal” through development (*see Methods* for criteria used for designations), which revealed a progressive increase in the percentage of abnormal tubules into adulthood (Figure 1J). Collectively, our data indicated that *Cbfb* is essential for fertility, and disruption of *Cbfb* leads to the breakdown in spermatogenesis during prepubertal development.

### CBFβ is expressed at multiple stages of spermatogenesis

**FIGURE 2:**
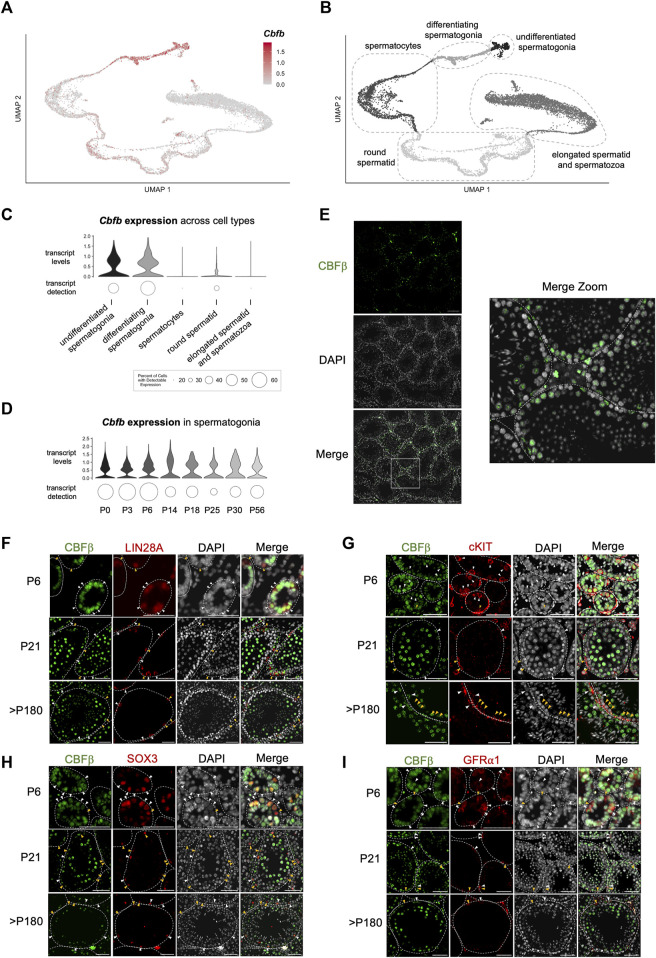
Expression of *Cbfb* throughout postnatal germline development. **(A–D)** Analysis of published scRNA-seq data from the postnatal germline development. Uniform manifold approximation projection (UMAP) representation of *Cbfb* transcripts **(A)** and germ cell subtypes **(B)** in the adult (P56) germline. Also, violin plots of *Cbfb* transcript abundance within germ cell subtypes **(C)** and spermatogonia through postnatal development **(D)** are included. **(E)** Representative images of immunofluorescence staining using an antibody recognizing CBFβ (green) and DAPI (grey) from testis cross-sections of adult wild-type mice. **(F–I)** Representative images from co-immunofluorescence staining for CBFβ (green) and select spermatogonia markers within the postnatal testis, including LIN28A [**(F)** undifferentiated spermatogonia], cKIT [**(G)** differentiating spermatogonia], GFR⍺1 [**(H)** SSC-enriched spermatogonia], and SOX3 [**(I)** progenitor-enriched spermatogonia]. White arrowhead identifies CBFβ+ cells. Yellow arrowhead identifies CBFβ-cell. Scale bar is 50 µm for images in **(E–I)**.

To gain greater insights into the functional role(s) of CBFβ within the various cell types of the male germline, we performed a series of gene expression analyses. First, we mined published single-cell RNA-sequencing (scRNA-seq) data of the postnatal germline (Green et al., 2018; Hermann et al., 2018; Grive et al., 2019; Law et al., 2019; Tan et al., 2020). In adult (P56) germ cells, *Cbfb* transcripts were heterogeneously detected among spermatogonia, late-stage spermatocytes, and round spermatid (Figures 2A–C; Supplementary Figures S2A–H). Furthermore, the level of *Cbfb* transcript abundance remained relatively constant through prepubertal development within each population, including, for example, spermatogonia (Figure 2D). Immunofluorescent staining of wild-type testis cross-sections largely confirmed these findings with protein expression of CBFβ localized to the same populations (Figure 2E).

Spermatogonia along the basement membrane are functionally heterogeneous and comprise either differentiating spermatogonia that have irreversibly committed to differentiation in response to a transient pulse of RA or constitute the replenishing population of undifferentiated spermatogonia that repopulate in preparation for the next RA pulse. Therefore, we performed co-immunofluorescent staining of CBFβ, and either the undifferentiated spermatogonia marker LIN28A or the differentiating spermatogonia marker cKIT (Figures 2F, G; Supplementary Figure S2I). Outcomes revealed that CBFβ co-localized with both LIN28A+ and cKIT + germ cells. Furthermore, within the undifferentiated spermatogonia population, we observed CBFβ expression within both GFRA1+ stem cell-enriched and SOX3+ progenitor-enriched subsets (Figures 2H, I). Collectively, scRNA-seq and immunostaining experiments indicated that CBFβ is broadly expressed within undifferentiated spermatogonia as well as differentiating spermatogonia, late-stage spermatocytes, and round spermatid.

### CBFβ functions within undifferentiated spermatogonia to support spermatogenesis

**FIGURE 3:**
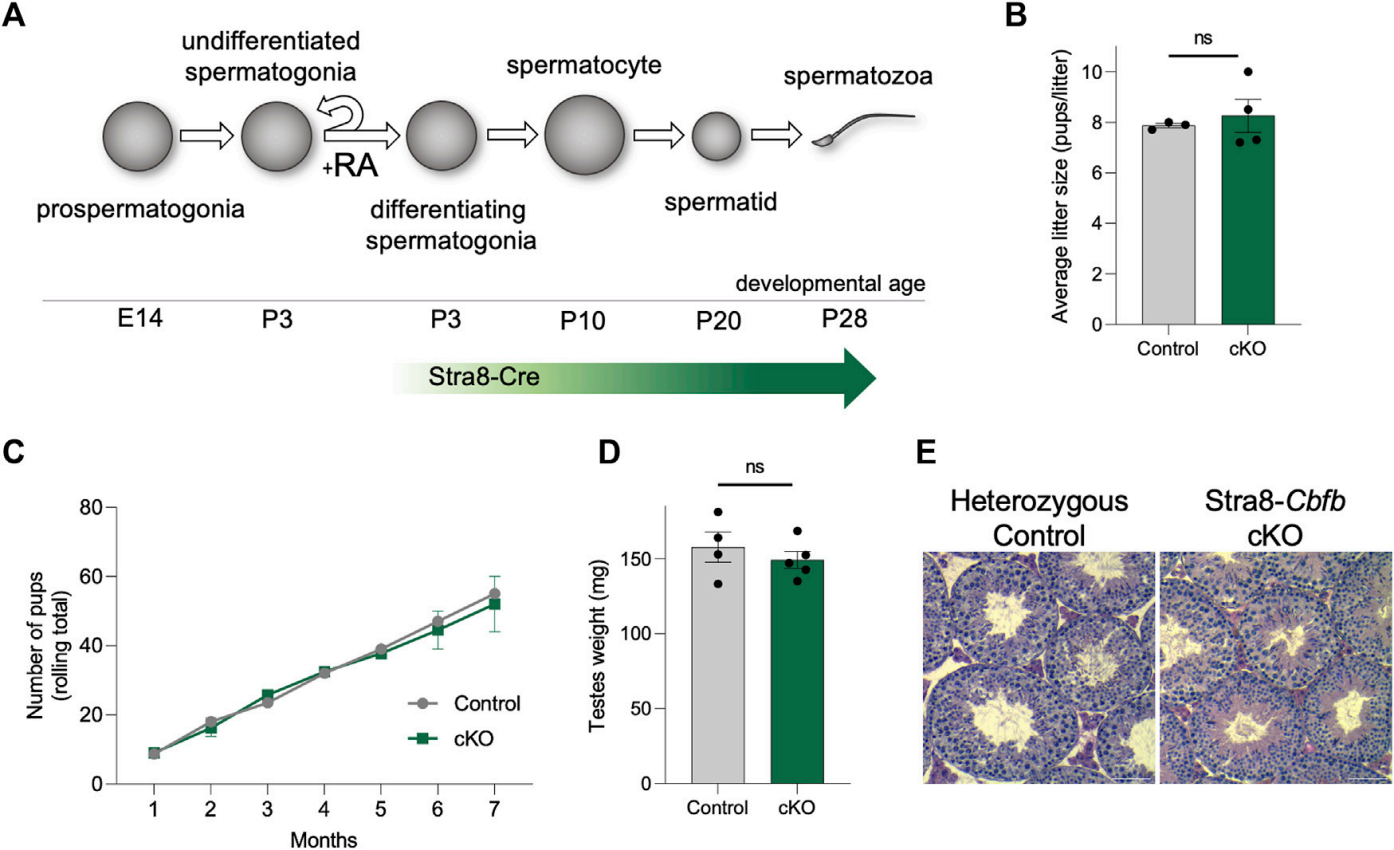
Inactivation of *Cbfb* in differentiating spermatogonia and subsequent germ cells via Stra8-Cre does not alter male fertility.**(A)** Schematic representation of Stra8-Cre activity in the male germline. **(B,C)** Average litter size **(B)** and cumulative number of pups **(C)** from heterozygous control and Stra8-*Cbfb* cKO males bred to wild-type females throughout the breeding trial. **(D,E)** Mean paired testis weight **(D)** and representative images from H&E staining of testis cross-sections **(E)** from heterozygous control and Stra8-*Cbfb* cKO males at the end of the breeding trial (>P180). Scale bar is 100 µm. Quantifications in **(B–D)** are presented as means ± SEM for *n* = 3–5 biologically independent animals per genotype and age. *p < 0.05, **p < 0.01, ***p < 0.001; not significant (ns).

Next, we utilized another genetic approach to better isolate the function of CBFβ within the various postnatal germ cell sub-types. To accomplish this, we inactivated *Cbfb* from differentiating spermatogonia and all subsequent germ cell sub-types using a Stra8-Cre^Tg^ mouse line (denoted Stra8-*Cbfb* cKO) (Figure 3A). Surprisingly, Stra8-*Cbfb* cKO male mice were fertile when bred to wild-type females during a 6-month breeding trial (Figures 3B, C). To confirm Cre excision within the *Cbfb* locus, progeny of Stra8-*Cbfb* cKO males from several litters were genotyped; all pups were heterozygous for the null allele (*Cbfb* ^Δ/wt^). Consistent with fertility outcomes, mean paired testis weights following the breeding trial were not statistically different between Stra8-*Cbfb* cKO and controls (Figure 3D), and Stra8-*Cbfb* cKO testes exhibited normal spermatogenesis based on histological evaluation (Figure 3E). Together, our Stra8-*Cbfb* cKO studies indicated that *Cbfb* expression in differentiating spermatogonia and advanced germ cell sub-types is not essential for germline function despite being expressed in these populations. However, paired with findings from Blimp1-*Cbfb* cKO mice that revealed extensive breakdown in spermatogenesis postnatally, our data indicated that the functional role of CBFβ is likely confined to the undifferentiated spermatogonia population.

### CBFβ regulates maintenance of undifferentiated spermatogonia

**FIGURE 4:**
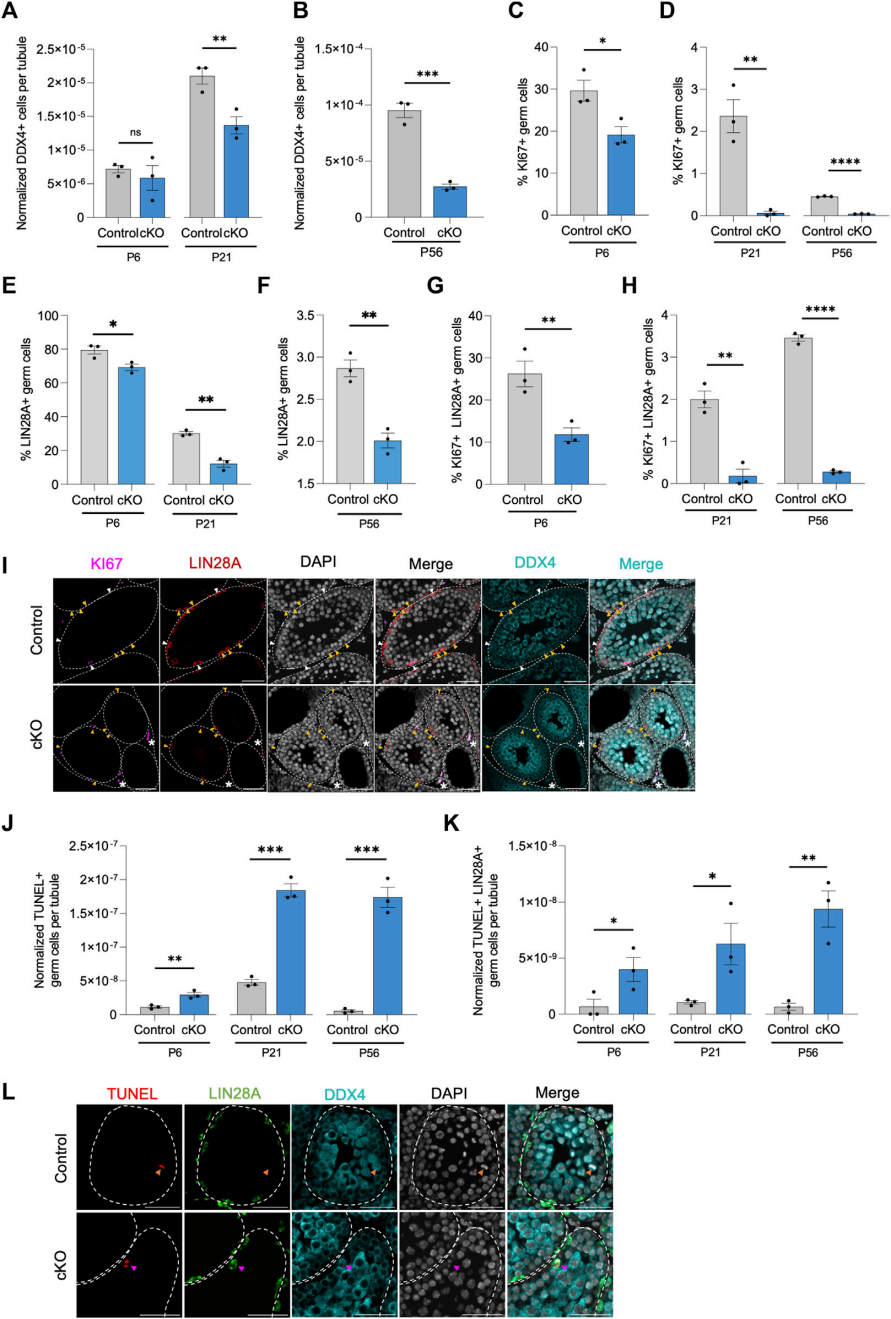
CBFβ is important for germline maintenance in the undifferentiated spermatogonia. **(A,B)** Quantification of DDX4+ germ cells per tubule cross-section. Values normalized to cross-sectional area. **(C–H)** Additional quantifications for the percent of KI67+ proliferative germ cell [KI67+ DDX4+; **(C,D)**], the percent of LIN28A + undifferentiated spermatogonia within all DDX4+ germ cells **(E,F)**, and the percent of proliferative undifferentiated spermatogonia [KI67+ LIN28A + DDX4+; **(G,H)**] from testis cross-sections of heterozygous control and Blimp1-*Cbfb* cKO. **(I)** Representative images from immunofluorescence staining of testis cross-sections at P21 using antibodies recognizing KI67 (magenta), LIN28A (red), and DDX4 (cyan) with DAPI (grey) in heterozygous control and Blimp1-*Cbfb* cKO. White arrowheads indicate proliferative undifferentiated spermatogonia (KI67+ LIN28A + DDX4+). Yellow arrowheads identify non-proliferative undifferentiated spermatogonia (KI67- LIN28A + DDX4+) cells. Asterisks identify KI67+ interstitial cells. **(J,K)** Quantifications of apoptotic germ cells [TUNEL+ DDX4+; **(K)**] and apoptotic undifferentiated spermatogonia [TUNEL+ LIN28A + DDX4+; **(K)**] per tubule cross-section normalized to cross-sectional area, of heterozygous control and Blimp1-*Cbfb* cKO throughout ages. **(L)** Representative immunofluorescence images of testis cross-sections at P21 with fluorescent signals corresponding to TUNEL (red), LIN28A (green), DDX4 (cyan), and DAPI (grey). Magenta arrowheads indicate TUNEL+ LIN28A + DDX4+ cells. Orange arrowheads indicate TUNEL+ LIN28A- DDX4+ cells. Scale bar is 50 µm for images in **(I,L)**. Quantifications in **(A–H)** and **(J,K)** are presented as means ± SEM for *n* = 3 biologically independent animals per genotype and age. *p < 0.05, **p < 0.01, ***p < 0.001; not significant (ns).

Following each pulse of RA which commits the majority of undifferentiated spermatogonia to differentiation, the remaining undifferentiated spermatogonia transiently amplify to replenish the population in preparation for the next pulse of RA stimulation. In doing so, the germline is maintained indefinitely, round after round. The absence of germ cells within some tubules of Blimp1-*Cbfb* cKO testes suggests that germline maintenance may be somehow disrupted. To better understand the function of CBFβ in germline maintenance, we quantified the number of DDX4+ germ cells per testis cross-section through development. Outcomes revealed that the overall number of germ cells within tubule cross-sections progressively decreased in Blimp1-*Cbfb* cKO testes (Figures 4A, B). We then quantified KI67+ proliferative germ cells in both Blimp1-*Cbfb* cKO and control testis cross-sections and observed significant reductions at P6, P21, and P56 (Figures 4C, D, I; Supplementary Figures 3A, B). Strikingly, proliferation among DDX4+ germ cells drastically decreased by 97.5% at P21 (*p* = 0.0043) and 90.7% at P56 (*p* < 0.0001). Focusing on the undifferentiated spermatogonia population, the percentage of LIN28A + germ cells was significantly reduced in Blimp1-*Cbfb* cKO testes compared to controls at P6, P21, and P56 (Figures 4E, F), with corresponding decreases in the percentage of KI67+ proliferative LIN28A + germ cells at each age (Figures 4G–I; Supplementary Figures 3C, D).

Decreased population sizes could be attributed to disrupted proliferation, cell death, or both. Therefore, we utilized TUNEL immunofluorescent staining to measure the number of apoptotic germ cells. Quantification of TUNEL analysis revealed an overall increase in apoptotic germ cells at P6, P21, and P56 in Blimp1-*Cbfb* cKO testis cross-sections compared to controls (Figures 4J, L). Similarly, we observed a significant increase in apoptotic LIN28A + germ cells with Blimp1-*Cbfb* cKO (Figures 4K, L). Collectively, these data pointed to the role of CBFβ in maintaining the undifferentiated spermatogonia population by regulating proliferation and survival through postnatal development and into adulthood.

### The role of CBFβ in meiotic completion

**FIGURE 5:**
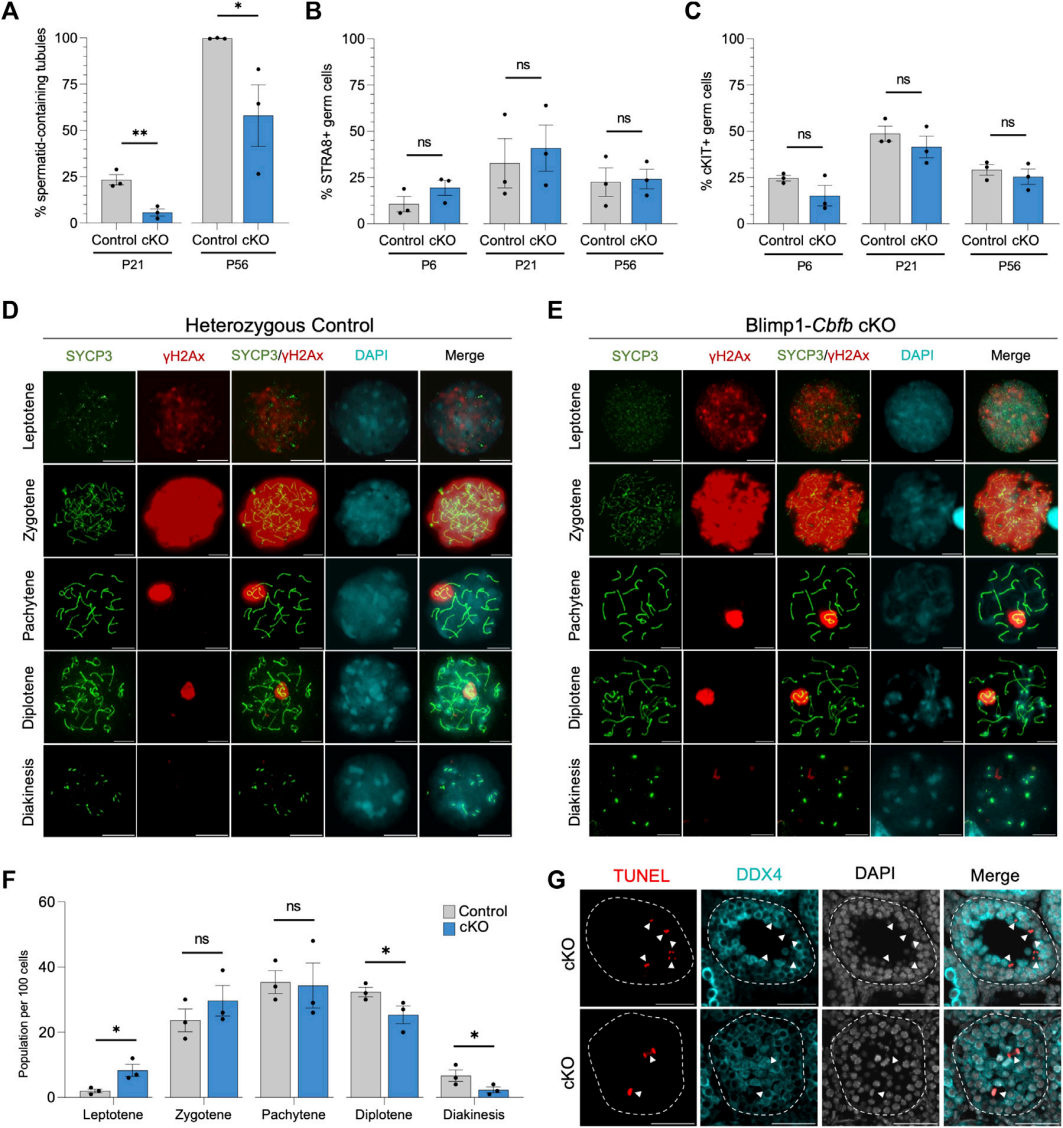
CBFβ is necessary for spermatid formation. **(A)** Quantification of percent seminiferous tubules that contain spermatid within testis cross-sections. **(B,C)** Quantification of differentiating spermatogonia population marked by either STRA8 **(B)** or cKIT **(C)** alongside DDX4. **(D,E)** Representative immunofluorescence images of P20 spermatocyte spreads from heterozygous control **(D)** or Blimp1-*Cbfb* cKO **(E)** males stained with antibodies recognizing SYCP3 (green), yH2Ax (red), and DAPI (cyan). Scale bar is 10 µm. **(F)** Quantification of prophase I meiotic stages in P20 heterozygous control and Blimp1-*Cbfb* cKO via spermatocyte spreads. **(G)** Representative immunofluorescence images of TUNEL staining (red) in advanced germ cells within P21 Blimp1-Cbfb cKO testis cross-sections using antibodies recognizing LIN28A (green) and DDX4 (cyan) with DAPI (grey). Scale bar is 50 µm. White arrowheads indicate TUNEL+ DDX4+ cells. Quantifications in **(A–C and F)** are presented as mean ± SEM for *n* = 3 biologically independent animals per genotype and age. *p < 0.05, **p < 0.01, ***p < 0.001; not significant (ns).

Our cKO studies indicated that *Cbfb* inactivation in undifferentiated spermatogonia had distal effects on germ cell maturation, including evidence of reduced spermatid and spermatozoa formation. Therefore, we quantified the number of seminiferous tubules containing round spermatid in Blimp1-*Cbfb* cKO and control testes using H&E cross-sections. Our analysis revealed a significant decrease in the percentage of tubules containing round spermatid at P21 and P56 in Blimp1-*Cbfb* cKOs compared to controls (Figure 5A).

Reduced spermatid formation and the presence of tubules containing only spermatogonia within Blimp1-*Cbfb* cKO may be attributed to the failure of germ cells to initiate terminal differentiation and form differentiating spermatogonia. However, immunofluorescent staining of testis cross-sections using antibodies recognizing cKIT and STRA8, markers of differentiating spermatogonia, revealed no significant changes in the percentage of either cKIT + or STRA8+ germ cells (Figures 5B, C) or the number of STRA8+ cells per tubule cross-section (Supplementary Figures 4A, B). Alternatively, reduced spermatid formation may point to deficits in meiotic progression. Quantification of meiotic stages in spermatocytes from Blimp1-*Cbfb* cKO and control testes using immunofluorescent staining for the synaptonemal complex marker SYCP3 and the DNA double-strand break marker yH2Ax revealed a significant increase in leptotene spermatocytes but a decrease in spermatocytes within diplotene and diakinesis stages (Figures 5D–F). Surprisingly, no observable differences in either synaptonemal complex composition or the representation of each meiotic stage differed between Blimp1-*Cbfb* cKO and control spermatocytes. However, this temporal shift in meiotic progression may lead to reduced spermatocytes completing meiosis. Consistently, we observed a higher frequency of TUNEL+ spermatocytes undergoing apoptosis (Figure 5G). Collectively, these experiments suggested that the actions of CBFβ within the undifferentiated spermatogonia population distally influenced meiotic progression and spermatid formation.

### CBFβ regulates transcriptional networks associated with proliferation

To gain further insights into the gene expression pathways downstream CBFβ, we utilized NanoString GeoMx Whole Transcriptomic Analysis (WTA) to identify differentially expressed genes (DEGs) between Blimp1-*Cbfb* cKO and control animals. We chose to analyze transcriptomes at P6, when the first cKO phenotypes appeared, and P21, when deficits in meiotic progression and spermatid formation were apparent. Two slides, one for P6 and one for P21, were prepared for WTA that contained triplicate testis cross-sections from 2–3 animals per genotype at each developmental age (*n* = 3 animals per genotype at P6, *n* = 2 animals per genotype at P21) to achieve both technical and biological replications. Due to size constraints in the scannable area of the NanoString Digital Spatial Profiler, only *n* = 2 animals per genotype could be captured for P21. Therefore, given the diminished statistical power, we identified DEGs based on both statistical significance (*p* < 0.05) and log2 fold change (log2FC). To isolate transcriptomic signatures from undifferentiated spermatogonia or terminally differentiating germ cells (i.e., differentiating spermatogonia, spermatocytes, and spermatid), we stained with antibodies recognizing LIN28A and DDX4 alongside the nuclear stain SYTO13. The resulting fluorescent signatures were then used to photoactivate precise regions within testis cross-sections to isolate transcriptomic probes within target populations.

**FIGURE 6:**
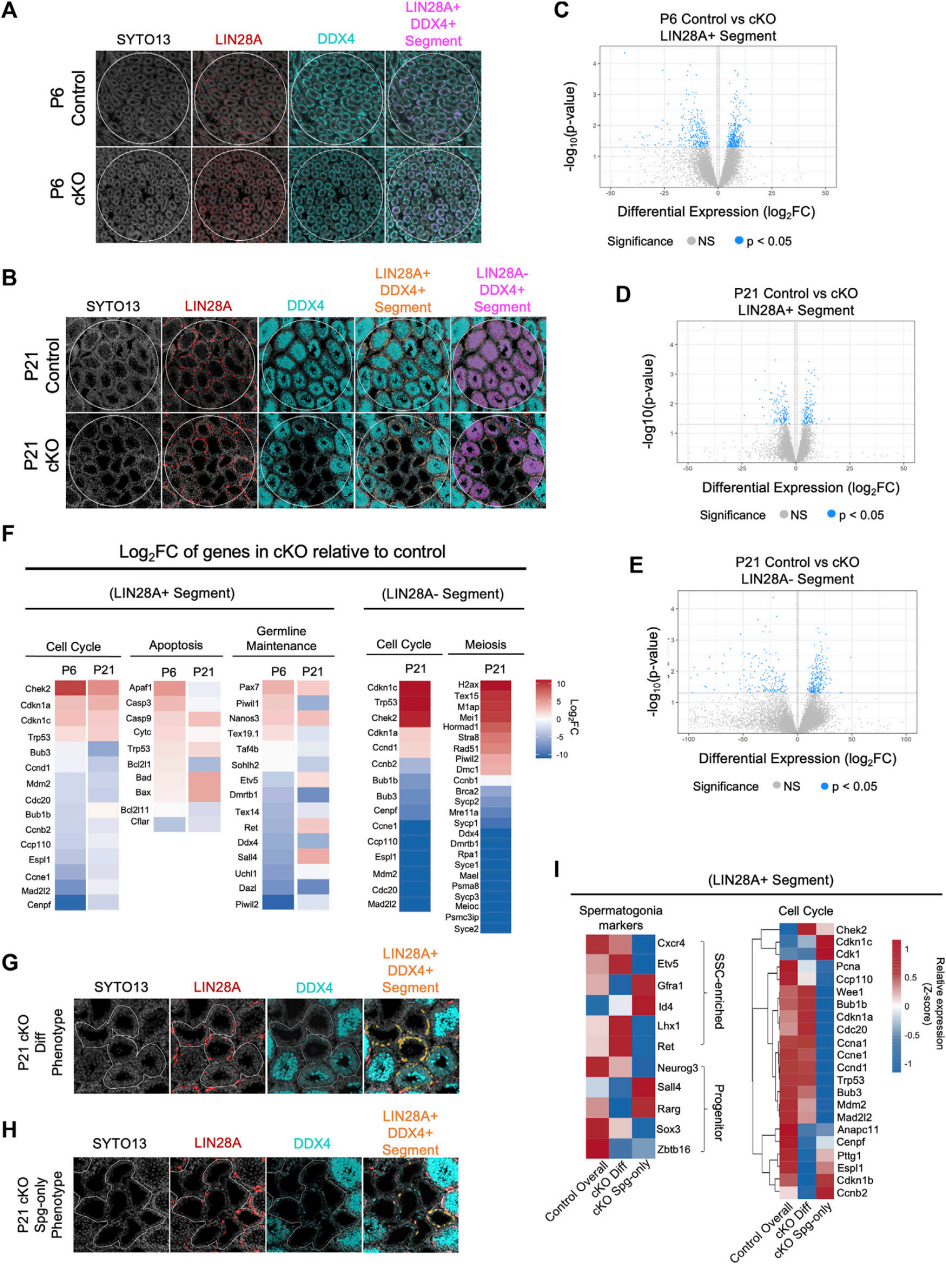
CBFB regulates cell cycle progression for germline maintenance and differentiation. **(A,B)** Representative images of immunofluorescence staining using SYTO13 (grey) as DNA stain, LIN28A (red), and DDX4 (cyan) with testis cross-sections from P6 **(A)** and P21 **(B)** heterozygous control and Blimp1-*Cbfb* cKO males for NanoString GeoMx spatial transcriptomics. Regions of interest (ROIs) are denoted with white circles and segmentation of target cell populations within each ROI is indicated in orange or magenta. **(C–E)** Volcano plots of DEGs within P6 LIN28A + DDX4+ SYTO13+ segments **(C)** and P21 LIN28A + DDX4+ SYTO13+ segments **(D)**, and P21 LIN28A- DDX4+ SYTO13+ segments **(E)**. **(F)** Heatmap representation of select DEGs as log2FC comparing Blimp1-*Cbfb* cKO with heterozygous control testes at P6 and P21 in both the LIN28A + DDX4+ SYTO13+ segments and at P21 in LIN28A- DDX4+ SYTO13+ segments. **(G,H)** Representative immunofluorescence images from capture of heterogeneous seminiferous tubule phenotypes in P21 Blimp1-*Cbfb* cKO, including regions with discontinuous spermatocyte development [Diff Phenotype; **(G)**] and tubules that contain only spermatogonia [Spg-only Phenotype; **(H)**]. Regions were segmented for LIN28A + DDX4+ fluorescent signal **(I)** Heatmap representation of relative expression values for select genes in the undifferentiated spermatogonia (LIN28A + DDX4+SYTO13+) within Diff phenotype and Spg-only phenotype ROIs.

Using this experimental design, we first performed a pseudo-bulk analysis to capture population-level *Cbfb-*dependent gene expression signatures within undifferentiated spermatogonia (P6, P21) and differentiating germ cells (P21). To accomplish this, we randomly placed one circular region of interest (ROI) on each tissue cross-section and segmented within each ROI based on LIN28A+ (LIN28A + DDX4+SYTO13+) or LIN28A-DDX4+ (LIN28A-DDX4+SYTO13+) fluorescent signals to enrich for the undifferentiated spermatogonia or differentiating germ cells, respectively (Figures 6A, B).

Within the P6 LIN28A + segment, we identified a total of 672 statistically significant DEGs (*p* < 0.05), or 10,542 DEGs with >2.0 log2FC, between Blimp1-*Cbfb* cKO and controls (Figure 6C). Within the P21 LIN28A + segments, we identified 282 significant DEGs (*p* < 0.05), or 5,622 DEGs with >2.0 log2FC (Figure 6D). Interestingly, 14 statistically significant DEGs (*p* < 0.05) were shared between the P6 and P21 ages, or 2,000 DEGs using a >2.0 log2FC cutoff (Supplementary Figures 5A, B). Gene ontology analysis of DEGs revealed downregulation of several genes involved in proliferation. In particular, genes encoding components of the mitotic checkpoint complex, including *Cdc20, Mad2l2, Bub3, Ccp110*, and *Espl1*, were downregulated at both P6 and P21. Additionally, pro-apoptotic genes (*Bad*, *Bax*, *Casp9*, and *Trp53*) were upregulated, and anti-apoptotic genes (*Bcl2l11* and *Cflar*) were downregulated in LIN28A + segments. Interestingly, genes implicated in SSC self-renewal, such as *Lhx1*, *Gfra1*, and *Id4*, were not differentially expressed with Blimp1-*Cbfb* cKO in the LIN28A + segments (Figure 6F).

Within the LIN28A-DDX4+ segments, differential gene expression analysis revealed 422 significant DEGs (*p* < 0.05), or 12,014 DEGs with >2.0 log2FC, with P21 Blimp1-*Cbfb* cKO compared to controls (Figure 6E). Gene ontology analysis of DEGs highlighted several downregulated biological processes associated with cell cycle regulation, sperm maturation/differentiation, and flagella- and cilia-mediated motility (Supplementary Figures 5C, D). Furthermore, numerous canonical meiotic regulators, such as *Sycp1*, *Sycp2*, *Sycp3*, *Syce1*, *Dmc1*, *Meioc*, and others, were downregulated with Blimp1-*Cbfb* cKO. Strikingly, many of the same cell cycle-related genes of the mitotic checkpoint complex that were downregulated in LIN28A + segments were similarly downregulated in LIN28A-DDX4+ segments, including *Bub1b*, *Bub3*, *Cdc20*, *Mad2l2*, *Ccp110*, and *Espl1* (Figure 6F). Significant downregulation of mitotic checkpoint control (*Bub1b*, *Bub3*, and *Mad2l2*) and meiotic (*Sycp1* and *Sycp3*) genes with Blimp1-*Cbfb* cKO was further validated via qPCR (Supplementary Figure 5E).

### CBFβ regulates discrete cell cycle checkpoint control programs

In the experiments above (Figure 1I), we observed phenotypic heterogeneity among discrete regions of the developing Blimp1-*Cbfb* cKO testis such that some tubule cross-sections contained only spermatogonia while others contained different degrees of breakdown during spermatocyte and spermatid maturation. Given that our findings indicated that CBFβ function is limited undifferentiated spermatogonia, we hypothesized that this phenotypic heterogeneity may coincide with functional differences among undifferentiated spermatogonia. To test this hypothesis, we utilized spatial transcriptomics to create ROIs around P21 Blimp1-*Cbfb* cKO tubule cross-sections grouped into two phenotypes: (Griswold, 2022): tubules in which germ cell maturation at spermatocyte or spermatid stages appeared discontinuous or disrupted (termed the differentiation phenotype or “cKO Diff Phenotype”) and (Raverdeau et al., 2012) tubules which contained only LIN28A + undifferentiated spermatogonia and no advanced germ cells (termed the spermatogonia-only phenotype or “cKO Spg-only Phenotype”; Figures 6G, H). As before, we then segmented these ROIs to isolate the LIN28A+ (LIN28A + DDX4+SYTO13+) regions to enrich for undifferentiated spermatogonia and performed differential expression analysis.

Surprisingly, spermatogonia markers that enrich for either SSCs (*Cxcr4*, *Etv5*, *Gfra1*, *Id4*, *Lhx1*, and *Ret*) or more progenitor-like undifferentiated spermatogonia (*Neurog3*, *Sall4*, *Rarg*, *Sox3*, and *Zbtb16*), showed mixed expression within the cKO Diff and cKO Spg-only tubule phenotypes; this suggested that tubule phenotypes were not correlated with functional fates within undifferentiated spermatogonia (Figure 6I). Interestingly, however, among checkpoint control genes, many interphase and mitotic complex-related genes (*Pcna*, *Ccnd1*, *Ccp110*, *Bub1b*, *Bub3*, *Cdc20*, *Mdm2*, and *Mad2l2*) were downregulated in the cKO Spg-only Phenotype while genes necessary for the completion of cell division (*Ccnb2*, *Cdk1*, *Cenpf*, *Pttg1*, and *Espl1*) were downregulate in cKO Diff Phenotype (Figure 6I). Thus, our data suggest that tubule phenotypes within Blimp1-*Cbfb* cKO testes coincide with discrete differences in *Cbfb-*dependent checkpoint control genes and different proliferative signatures among the undifferentiated spermatogonia population.

## Discussion

Studies have highlighted the significance of CBFβ in development and homeostasis across many tissues, such as bone, blood, brain, hair, and skin (Speck et al., 1999; Komori, 2003; Inoue et al., 2008; Link et al., 2010; Kurosaka et al., 2011; Tian et al., 2014; Qin et al., 2015; Mevel et al., 2019). Here, our study demonstrated the essential role of *Cbfb* in the male germline. CBFβ is expressed in several germ cell types throughout the stages of spermatogenesis. Therefore, we generated Blimp1-*Cbfb* cKO model to ablate *Cbfb* from the germline throughout prenatal and postnatal testis development to explore function. Using the Blimp1-*Cbfb* cKO model, we discovered that *Cbfb* is necessary for sperm production, sperm motility, and overall male fertility. Additionally, significant reductions in testis weight and intra-testicular sperm production were observed starting prior to puberty and sustained into adulthood. Together, our study highlights the important role of CBFβ during prepubertal germline development, which is necessary to establish and maintain spermatogenesis long into adulthood.

Most undifferentiated spermatogonia respond to a transient spike in RA levels amid the seminiferous cycle and initiate terminal differentiation; those undifferentiated spermatogonia that do not, proliferate to replenish the undifferentiated spermatogonia population in preparation for the next RA pulse (Tegelenbosch and Rooij, 1993; Griswold, 2016). This process of committing germ cells to differentiation via RA and rebuilding the undifferentiated spermatogonia population repeats with each spermatogenic cycle to ensure continuous production of differentiating spermatogonia while maintaining the undifferentiated spermatogonia population indefinitely (de Rooij and Russel, 2000). On one hand, the production of differentiating spermatogonia was not impacted in Blimp1-*Cbfb* cKO animals, as evidenced by no significant change in the STRA8+ or cKIT + germ cell populations. On the other hand, proliferation from the undifferentiated spermatogonia population was profoundly reduced in undifferentiated spermatogonia, accompanied by a significant increase in apoptosis. Through prepubertal development, the undifferentiated spermatogonia population gradually declined in Blimp1-*Cbfb* cKO testes and seminiferous tubules progressively became devoid of germ cells. These observations demonstrated that CBFβ is critical for germline maintenance. This failed maintenance could be attributed to either disruption in SSC self-renewal or undifferentiated spermatogonia that attempted to proliferate and were instead lost to programmed cell death via apoptosis. However, further experiments such as with SSC transplantation are necessary to assess CBFβ regulation of self-renewal more directly. Regardless, the rapid demise of the germline within *Cbfb* inactivation underscores the central role of CBFβ in germline maintenance. Furthermore, during this window of prepubertal development, undifferentiated spermatogonia rapidly proliferate to build the population in preparation for adult spermatogenesis (Drumond et al., 2011; Law et al., 2019). With the decline of the undifferentiated spermatogonia population in Blimp1-*Cbfb* cKO testes, our data indicate that CBFβ is necessary for establishment and maintenance of the undifferentiated spermatogonia population.

Utilizing complementary genetic approaches with two different Cre drivers, our studies determined that the functions of *Cbfb* in spermatogenesis are most likely confined to the undifferentiated spermatogonia population. Blimp1-Cre is active prenatally in PGCs and prospermatogonia (Ohinata et al., 2005; Yamashiro et al., 2016). Therefore, we cannot rule out the potential that PGC and/or prospermatogonial development were impacted with Blimp1-*Cbfb* cKO. However, at birth, Blimp1-*Cbfb* cKO testes were phenotypically normal, suggesting that prenatal germline development was not impacted by *Cbfb* inactivation. By contrast, the first evidence of germline disruption in Blimp1-*Cbfb* cKO testes appeared at P6, at which point most germ cells have transitioned to form spermatogonia (Drumond et al., 2011; McCarrey, 2013). The neonatal spermatogonia population is composed of either undifferentiated or differentiating spermatogonia, labeled by LIN28A and STRA8, respectively. To parse out the function of *Cbfb* in differentiating spermatogonia and advanced germs (i.e., spermatocytes, spermatid, and spermatozoa) derived from undifferentiated spermatogonia, we utilized Stra8-Cre to generate a different cKO model. Outcomes revealed that Stra8-*Cbfb* cKO males were fertile with no observable disruption to spermatogenesis, thus demonstrating that CBFβ within the differentiating spermatogonia and all subsequent germ cell sub-types is dispensable. However, inactivation of *Cbfb* within undifferentiated spermatogonia via Blimp1-Cre led to multi-stage breakdown in the male germline and sterility. Collectively, evaluating both cKO models narrows the functional role of CBFβ to likely be within the undifferentiated spermatogonia population.

In addition to regulating germline maintenance, our experiments indicate that the inactivation of *Cbfb* in undifferentiated spermatogonia impacted meiotic completion. Analysis of Blimp1-*Cbfb* cKO testes revealed no significant change in the STRA8+ and cKIT + germ cell populations, which indicates that entry to differentiation was not disrupted. However, further analysis of Blimp1-*Cbfb* cKO testes indicated a significant increase in leptotene spermatocytes and a decrease within diplotene and diakinesis stages of meiotic progression. This temporal disruption during meiotic progression in Blimp1-*Cbfb* cKO testes likely reduced the number of spermatocytes completing meiosis and forming spermatid (Gorbsky, 2015). Consistently, we observed elevated apoptosis in spermatocytes in Blimp1-*Cbfb* cKO testes and transcriptomic studies revealed downregulation of canonical meiotic genes. In addition, our transcriptomic studies revealed that many of the cellular components necessary for sperm motility and function were significantly downregulated with *Cbfb* inactivation, which may, in part, underlie the impaired sperm motility of Blimp1-*Cbfb* cKO males. However, downregulation of genes implicated in sperm motility may also reflect the temporal shifts in meiotic progression and spermatid formation. Given that disruptions in prepubertal germline development only occur with *Cbfb* inactivation in undifferentiated spermatogonia, our findings suggest that CBFβ is necessary to set up molecular frameworks in undifferentiated spermatogonia that directly or indirectly influence later stages of terminal differentiation, such as meiosis and sperm maturation.

Here, we shed light on the functional roles of CBFβ in regulating both proliferation and differentiation concurrently during postnatal germline development. How is CBFβ able to modulate both processes simultaneously? To start addressing this question, we used spatial transcriptomics and, surprisingly, discovered that key cell cycle checkpoint genes were downregulated within both undifferentiated spermatogonia and advanced germ cells of Blimp1-*Cbfb* cKOs, which suggests that CBFβ may regulate both proliferation and differentiation by modulating cell cycle control within these processes. For example, genes of the mitotic checkpoint complex, such as *Bub1* (or *Bub1b*), *Bub3*, *Cdc20*, and *Mad2* (or *Mad2l2*), were downregulated in both undifferentiated spermatogonia and advanced germ cells. The mitotic checkpoint complex is a well-established regulator of mitosis and numerous studies highlight its role in meiosis as well (Gorbsky, 2015; Liu and Zhang, 2016; Bolcun-Filas and Handel, 2018). Specifically, the mitotic checkpoint complex participates in proper chromosomal segregation during meiosis. Consistent with this, chromosomal synapsis and the early stages of meiosis were generally unaffected in Blimp1-*Cbfb* cKO males, but later stages of meiosis were significantly altered and overall spermatid formation following meiosis was blunted. Thus, how CBFβ accomplishes cell cycle control may fall within a common core proliferative program. However, the precise mechanism by which CBFβ exerts its diverse role using a common mechanism of action remains for future studies.

The ability of CBFβ to broadly regulate multiple aspects of germ cell maturation from within the undifferentiated spermatogonia population is both surprising and intriguing. Somehow, the distal programs for germ cell maturation, such as meiosis, are established within undifferentiated spermatogonia and carry indirectly to more advanced germ cells. However, an open question that remains is how is this programming accomplished? A recent study found that during the transition from mitosis to meiosis, there is a widespread reorganization of super-enhancers in the germline (Maezawa et al., 2020). However, within some locations of the genome, enhancers and suppressors related to mitosis and meiosis share similar epigenetic profiles (Maezawa et al., 2020). Thus, this potential programming from within the undifferentiated spermatogonia population may have epigenetic underpinnings. Studies suggest that, in addition to RUNX proteins, CBFβ can form heterodimeric complexes with histone modifiers (Bruno et al., 2009; Roudaia et al., 2009; Mandoli et al., 2014). Therefore, the potential role of CBFβ in shaping the germline epigenome and/or establishing the meiotic program in undifferentiated spermatogonia warrants further investigation.

Collectively, our studies reveal multiple roles of CBFβ in prepubertal development within the male germline. From within the undifferentiated spermatogonia population, CBFβ regulates core frameworks that intersect different facets of germline maturation that when disrupted, lead to adult infertility.

## Materials and methods

### Animals

All procedures for the care and use of animals were approved by the Washington State University Animal Care and Use Committee. To generate Blimp1-Cre *Cbfb* cKO mice, male Blimp1(Prdm1)-Cre^Tg^ (Jackson Laboratories, stock no. 008827) and female *Cbfb* ^*fl/fl*^ (Jackson Laboratories, stock no. 028550) transgenic mice were first bred to generate Blimp1-Cre^Tg^;*Cbfb* ^*fl/+*^ animals. Then, female *Cbfb* ^*fl/fl*^ mice were crossed to Blimp1-Cre;*Cbfb* ^*fl/+*^ males to generate either Blimp1-Cre *Cbfb* cKO (Blimp1-Cre^Tg^;*Cbfb* ^*fl/fl*^) or heterozygous control (Blimp1-Cre^Tg^;*Cbfb* ^*fl/+*^) males. Stra8-Cre *Cbfb* cKO males were generated by first crossing female *Cbfb* ^*fl/fl*^ mice with male Stra8-Cre^Tg^ mice (Jackson Laboratories, stock no. 017490) to generate Stra8-Cre^Tg^;*Cbfb* ^*fl/+*^ animals. Female *Cbfb* ^*fl/fl*^ mice were then mated to Stra8-Cre^Tg^;*Cbfb* ^*fl/+*^ males to generate either Stra8-Cre *Cbfb* cKO (Stra8-Cre^Tg^;*Cbfb* ^*fl/fl*^) or heterozygous control (Stra8-Cre^Tg^;*Cbfb* ^*fl/+*^) males. During breeding trials, cKO and heterozygous control males were mated to wild-type C57BL/6J females for 4–6 months. Identification of a copulatory plug signified embryonic day 0.5 of gestation.

### Sperm and hormone analyses

Systemic blood from animals was collected and centrifuged at 2,000 xg for 15 min to isolate sera. Collected sera were sent to Ligand Assay and Analysis Core of the Center for Research in Reproduction at the University of Virginia for hormone analysis. Caudal epididymides were collected and teased apart in M2 medium (Sigma, M7167) and incubated for 15 min at 37°C to release sperm. Sperm concentration and motility were analyzed using Microptic computer-assisted sperm analysis (CASA) systems and Microptic SCA software (v. 6.4.0.82).

### Testis histology and immunostaining of testis cross-sections

Testes were collected detached from the epididymis prior to weight measurement. Collected testes were fixed in either Bouin’s solution (Electron Microscopy Sciences, 15,990) or 4% paraformaldehyde/PBS (Thermo Scientific™, 28,908) at room temperature or 4°C, respectively, for specified timeframes depending on the animal’s age (Barnard and Aston, 2013). Bouin and paraformaldehyde-fixed tissues were rinsed three times in 70% EtOH overnight at room temperature (RT) and PBS for 1 h, respectively. Isolated tissues were dehydrated through a graded ethanol series and xylenes washes prior to paraffin embedding. Paraformaldehyde-fixed paraffin-embedded (FFPE) and Bouin-fixed paraffin-embedded (BFPE) blocks of tissue were sectioned of 5 μm and deparaffinized in a graded series of xylenes and ethanol washes to generate testis cross-sections slides for histology, immunohistochemistry (IHC), and immunofluorescence (IF). For histology, BFPE testis cross-sections were stained with hematoxylin (Ricca Chemical, R3530000) and eosin Y (VWR, 95057-848). Following sodium citrate antigen retrieval, slides were then washed with 1X PBS three times at RT and incubated for 1 h in blocking buffer (1% serum matched to the secondary host species, 0.1% fish skin gelatin (Sigma, G7041), and 1X PBST (PBS + 0.01% Tween20) in a humidity chamber.

For IHC, ABC-peroxidase kit (Vector Laboratories, PK-6100) and DAB HRP Substrate Kit (Vector Laboratories, SK-4100) were used for immuno-visualization and followed with hematoxylin. Histology and IHC slides were mounted using VectaMount™ AQ Aqueous Mounting Medium (Vector Laboratories, H-5501-60), and images were taken using light microscopy (Nikon Eclipse E200; LasX software v.3.7.4) at 20X or 40X magnifications.

For IF, slides were briefly submerged in 0.1% Sudan black/EtOH to mitigate background autofluorescence, washed in 1X PBS, and incubated with select primary antibodies (*see* Table 1 for antibody details) in a humidified chamber overnight at 4°C. The next day, slides were washed three times in 1X PBS and incubated with the appropriate secondary antibody diluted in blocking buffer within a humidified chamber for 2 h at RT. Slides were washed three times in 1X PBS prior to mounting with Vectashield HardSet reagent containing DAPI (Vector Laboratories, H-1200-10). Slides were imaged using a DMi8 inverted fluorescent microscope (Leica-Microsystems; LasX v3.7.5) at 20X or 40X magnifications.

**TABLE 1:** Information pertaining to antibodies used in this study.

Antibody	Vendor and Cat. #	Application	Concentration
Rabbit anti CBFβ	Novus Biologicals, NBP1-87300	IF	1:200
Goat anti LIN28A	R&D Systems, AF3757	IF, NanoString	IF 1:250 NanoString 1:125
Goat anti cKIT	R&D Systems, AF1356	IF, IHC	1:125
Rabbit anti STRA8	Michael Griswold, WSU	IHC	1:200
Rat anti TRA98	Abcam, ab82527	IF	1:500
Rabbit anti DDX4	Abcam, ab13840	IHC, IF, NanoString	IF/IHC 1:200 NanoString 1:100
Rat anti Ki-67, FITC	Invitrogen, 11569882	IF	1:250
TUNEL	Roche, 12156792910	IF	—
Goat anti Rabbit IgG—Peroxidase (HRP)	Cell signaling, 7074	IHC	1:500
Donkey anti Rabbit Alexa Fluor 488	Invitrogen, A21206	IF, NanoString	IF 1:500 NanoString 1:500
Donkey anti Goat Alexa Fluor 555	Invitrogen, A21432	IF, NanoString	IF 1:500 NanoString 1:250
Donkey anti Rat Alexa Fluor 546	Invitrogen, A11081	IF	1:500
Donkey Serum	EMD Millipore, S30	IF, IHC	—
Goat Serum	EMD Millipore, S26	IF, IHC	—

### Single-cell RNA-sequencing data analysis

Single-cell transcriptomic data of the mouse male germline corresponding to ages P0, P3, P6, P14, P18, P25, P30, and P56 was acquired from the Gene Expression Omnibus (GEO; accession no. GSE124904, GSE121904, GSE109049, and GSE109033). Briefly, aligned and demultiplexed transcriptomes were computationally merged and assembled in R software using the Seurat package. Low-quality transcriptomes with >25% mitochondrial reads and >500 detected genes per cell were omitted. The aggregated data was then normalized, scaled, and dimensionally reduced via principal component analysis (PCA) and uniform manifold approximation and projection (UMAP) using default parameters and 50 principal components. Germ cells were isolated from within the data aggregate based on localized clustering and *Ddx4* transcript abundance. For gene expression analysis in Figure 2A; Supplementary Figure 2A, germ cells from P56 libraries were isolated and assembled with batch correction via fastMNN. Germ cell types were approximated based on marker genes identified by Hermann et al., 2018 (a sample of marker genes presented in Supplementary Figure 2A).

### TUNEL analysis

For terminal deoxynucleotidyl transferase dUTP nick end labeling (TUNEL) staining, IF slides following secondary antibody incubation were washed three times in 1X PBS. TUNEL solution (60 uL) was added to each slide and plastic coverslip was used to as slide cover during the 1 h incubation period at RT within a humidified chamber. Slides were washed following TUNEL incubation and mounted in similar manner as IF slides.

### Spermatocyte spread

Spermatocyte spreads were prepared according to previously published methods (Peters et al., 1997; Horan et al., 2017). Briefly, denunciated testes were first incubated in hypo-extraction buffer for 20 min before maceration in sucrose buffer using hypodermic needles to create a cell suspension. The resulting cell suspension was spread across 1% paraformaldehyde-coated slides and incubated in the humidified chamber for 2 h before air drying for 30 min. Dried slides were washed in 0.4% Photo-flo 200 solution for 2 min prior to long-term storage at −20°C. Preserved slides were thawed at RT for 1–2 min, incubated with primary antibody diluted in blocking buffer within a humidified chamber for 2 h at RT, washed three times in 1X PBS, and incubated with secondary antibodies diluted in blocking buffer in a humidified chamber for 2 h at RT. Slides were then washed thrice in PBS before mounting with a coverslip using Vectashield HardSet reagent containing DAPI. Slides were imaged using a DMi8 inverted fluorescent microscope at 60X magnification. For quantification of meiotic stages, 100 randomly selected spermatocytes were staged per animal.

### Quantification and statistical analyses

Tubules were counted for histology and IHC quantifications. Normal and abnormal seminiferous tubule criteria were determined using DDX4 as a germ cell marker via IHC. In P6, a tubule was considered normal if it had at least one germ cell, whereas the absence of a germ cell indicated an abnormal tubule. For P21, a normal tubule had spermatogonia and spermatocyte layers, while abnormal tubules lacked germ cells or had no spermatocytes. Observation of normal tubules in In P56 and >P180, normal tubules showed organized germ cell layers and spermatozoa (stage-dependent), while abnormal tubules lacked germ cells and had disorganized germ cell layers, including a lack of spermatids and spermatocytes. Fiji software (ImageJ software; v1.0) was used to quantify the different germ cell populations by applying a set threshold per antibody. Specific germ cell sub-populations were normalized to the number of tubules or overall germ cell populations using TRA98 or DDX4. Spermatid-containing tubules were quantified from histology images. All quantifications from testis cross-section were done on at least three non-adjacent, randomly selected sections per animal. Quantification of cell number per tubule was normalized to each individual testis cross-section area (mm^2^) that was measured through Fiji software (ImageJ software; v1.0). Statistical analysis of all quantitative comparisons between control and cKO was determined via *t*-test in Prism software (GraphPad Software; v.9.1.0). Data are presented as mean ± standard error of the mean (SEM). Each data point (n) represents an individual animal.

### qPCR analysis

RNA from FFPE testis cross-sections were extracted using RNeasy DSP FFPE Kit (Qiagen, 73604) using prepared 40 μm section (8 × 5 μm sections) of FFPE testis cross-sections per animal. For each animal, 1 μg of RNA was reverse transcribed to cDNA using QuantaBio qScript™ cDNA Synthesis Kits (VWR, 101414-098) on the same day as the RNA extraction. Once cDNA was generated, we performed qPCR using Applied Biosystems™ SYBR™ Green PCR Master Mix (Fisher Scientific, 43-091-55). qPCR analysis was done on P21 heterozygous control and Blimp1-*Cbfb* cKO (*n* = 3 per animals per genotype) using *Cbfb* primers (FP: TCG​AGA​ACG​AGG​AGT​TCT​TCA​GGA, RP: AGG​CGT​TCT​GGA​AGC​GTG​TCT), *Bub1b* primers (FP: GGA​AGA​CAA​TCA​GCC​CGG​AA, RP: CCC​CAG​ACC​AGT​TCT​TCA​CC), *Bub3* primers (FP: CGC​TTC​CCT​TGC​CTT​CAG​TA, RP: GGG​CTT​TGT​TTC​TGC​GTC​TG), *Mad2l2l* primers (FP: ATT​CTC​TAT​GTG​CGC​GAG​GTC, RP: TCC​AGG​AGA​GGT​TTG​ACG​CA), *Sycp1* primers (FP: CAA​AAG​CCC​TTC​ACA​CTG​TTC​G, RP: GTT​TTC​CCG​ACT​GGA​CAT​TGT​AA), and *Sycp3* primers (FP: AGC​CAG​TAA​CCA​GAA​AAT​TGA​GC, RP: CCA​CTG​CTG​CAA​CAC​ATT​CAT​A). *Rps2* primers (FP: CTG​ACT​CCC​GAC​CTC​TGG​AAA, RP: GAG​CCT​GGG​TCC​TCT​GAA​CA) were used as an internal control. qPCR reactions were performed using qTOWER^3^/G (Analytik Jena) and qPCRSoft software (v. 1.1.3.0).

### NanoString GeoMx spatial transcriptomics analysis

Spatial transcriptomic signatures of Blimp1-Cre *Cbfb* cKO and control testes were assayed using NanoString Whole Transcriptomic Analysis (WTA) kits (NanoString Technologies, GeoMx NGS RNA WTA Mm) from FFPE tissue blocks. For P6, *n* = 3 per animals per genotype were assayed. Due to size limitations within the collection area, *n* = 2 per animals per genotype were collected for P21. Sections from each tissue were generated at 5 μm thickness and slides were prepared according to NanoString GeoMx NGS FFPE manual slide preparation protocol with minor modifications. After sectioning the tissues, slides were incubated at 60°C for 30–60 min. Subsequently, deparaffination and hydration steps were carried out by incubating the slides sequentially for 5 min in xylene three times, 100% EtOH two times, 95% EtOH one time, and 1X PBS one time. To retrieve the target antigen, hydrated slides were briefly placed in 95°C DEPC H_2_O for 10 s, followed by immersion in a glass coplin jar containing 99°C–100°C 1X Tris-EDTA pH 9.0 in a high-temperature bead bath for 20 min. Slides then washed in 1X PBS for 5 min and incubated in 0.1 ug/mL proteinase K for 15 min at 37°C bead bath. Then, slides undergone series of 5 min washes for post fixation steps using 1X PBS one time, 10% neutral buffered formalin (NBF) one time, NBF stop buffer (0.1M Tris EDTA, 0.01M Glycine) two times, and another 1X PBS one time. For *in situ* hybridization, 200 μL of Buffer R, 25 μL of WTA probe mix, and 25 μL of DEPC H2O were added per slide. The slides were covered with a glass coverslip and incubated overnight in a humidity chamber at 37°C. The next day, slides were briefly submerged in 2X saline-sodium citrate buffer (SSCB) allowing coverslips to slide off. Then, off-target probes were removed using stringer wash solution (equal part formamide and 4X SSCB) twice at 37°C for 25 min each, followed by 2X SSCB washes two times for 2 min each. Blocking step was done by adding 200 uL of Buffer W per slide and slides were placed inside a humidity chamber for 30 min.

Following the blocking step, Buffer W was removed from the slide and replaced with unconjugated primary antibodies and DNA stain mixture that was diluted to the appropriate concentration using Buffer W. Primary antibody incubation was done to the slide in a humidity chamber for 1 h at RT. The slide was then quickly washed with 2x SSCB twice, followed by 3 min 2x SSCB washes three times. Slides were then incubated with secondary antibodies diluted in Buffer W for 30 min at RT in a humidified chamber. Slides were then washed 2x in SSCB buffer and directly imaged for RNA collection using a NanoString GeoMx Digital Spatial Profiler (DSP) and GeoMx DSP Control center software (v. 3.0.0.109). For overall gene expression signatures between control and cKO, three circular regions of interest (ROIs) of approximately 600–650 μm in diameter were randomly selected from three different cross-sections of each animal for technical replication. For phenotype-specific regions, tubules were grouped based on containing only a single layer of spermatogonia or containing an incomplete compliment of spermatocytes within the tubule cross-sections. ROIs were further segmented to capture undifferentiated spermatogonia or advanced germ cell (i.e., spermatocyte) populations separately by thresholding fluorescent signal corresponding to SYTO13+LIN28A + DDX4+ and SYTO13+LIN28A-DDX4+ regions within each ROI. A minimum of 50 nuclei were captured within each segmented population. After photo-activated probe collection, cDNA libraries were generated following the manufacturers protocol. Resulting libraries were pooled and sequenced in a single lane on an Illumina NovaSeq S4 lane (NovoGene Corporation, Inc.).

Raw sequencing reads were demultiplexed and probe counts corresponding to each gene were quantified using the NanoString GeoMX NGS Pipeline (v. 2.3.3.10). Count matrices were then analyzed in R software (v. 4.3.0) using the GeomxTools package (v. 3.4.0). Low-quality libraries were omitted from downstream analysis by selecting libraires with a Gene Detection Rate >= 1–5%. For both P6 and P21 datasets, a log_2_ fold change > 1.5 was used to define differentially expressed genes between groups as opposed to *p*-value because statistical strength was diminished by *n* = 2 biological replicates for P21 samples. Gene ontology was performed using clusterProfiler (v. 4.8.1) and PANTHER gene analysis (v. 17.0).

## Data Availability

The original contributions presented in the study are included in the article/Supplementary Material, further inquiries can be directed to the corresponding author.
