# Supplementary material for: Core binding factor subunit β plays diverse and essential roles in the male germline
Source: Front Cell Dev Biol. 2023 Nov 2;11:1284184. doi: 10.3389/fcell.2023.1284184 (PMC10653448; doi:10.3389/fcell.2023.1284184)
Supplement: Supplementary file 1 [file Presentation1.pdf]

Supplementary Figure 1 (connected to Fig. 1)

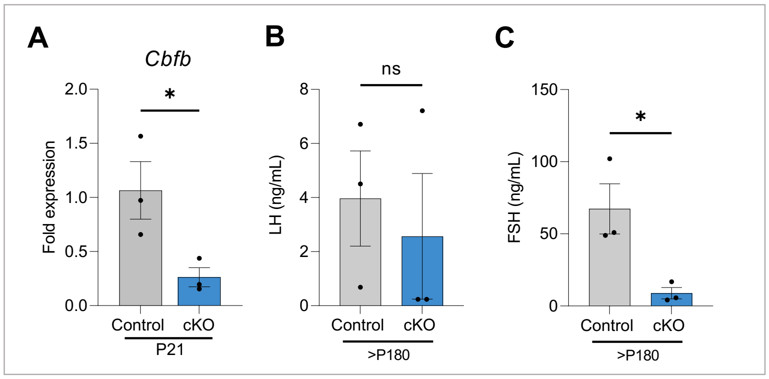

Supplementary Figure 1. **Blimp1-*Cbfb* cKO postnatal analyses.** (**A**) Fold expression of *Cbfb* expression using qPCR of heterozygous control and Blimp1-*Cbfb* cKO testes cross-sections. (**B-C**) Serum hormone analysis of LH (B) and FSH (C) levels of heterozygous control and Blimp1-*Cbfb* cKO animals following the breeding trial. Quantifications in A-C are presented as mean ± SEM for n = 3 biologically independent animals per genotype and age. *p<0.05; not significant (ns).

Supplementary Figure 2 (connected to Fig. 2)

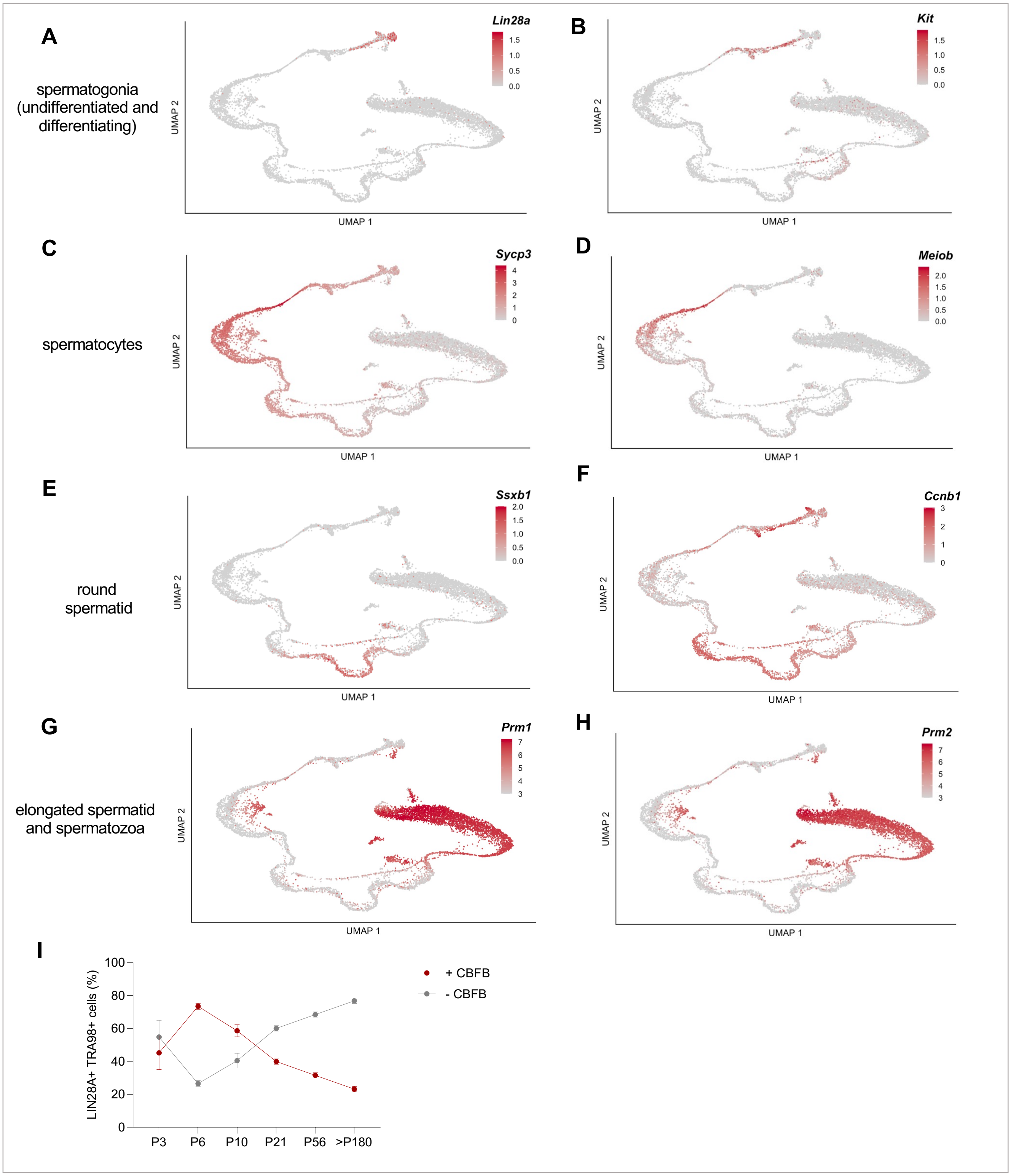

Supplementary Figure 2. **Expression of *Cbfb* within male germline.** (**A-H**) A sample of marker genes used to approximate each germ cell types in scRNA-seq transcriptomic analysis. (**I**) Quantification of CBFβ expression during postnatal germline development in the undifferentiated spermatogonia population using immunofluorescence staining of testis cross-sections using antibodies recognizing CBFβ, LIN28A, and TRA98. Data in I are presented as mean $\pm$ SEM for n = 3 biologically independent animals per age.

Supplementary Figure 3 (connected to Fig. 4)

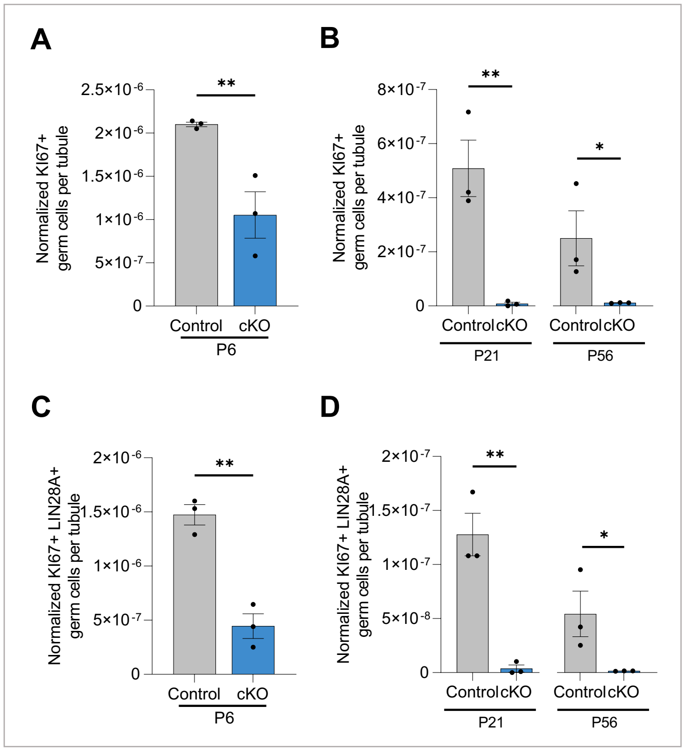

Supplementary Figure 3. Alternative quantification of germline proliferation with Blimp1-*Cbfb* cKO. (**A**-**D**) Quantification of KI67+ DDX4+ proliferative germ cells (A,B) and KI67+ proliferative LIN28A+ germ cells (C,D) per tubule cross-section. Values are normalized to testis cross-sectional area and presented as mean ± SEM for n = 3 biologically independent animals per genotype and age. *p<0.05, **p<0.01, ***p<0.001.

Supplementary Figure 4 (connected to Fig. 5)

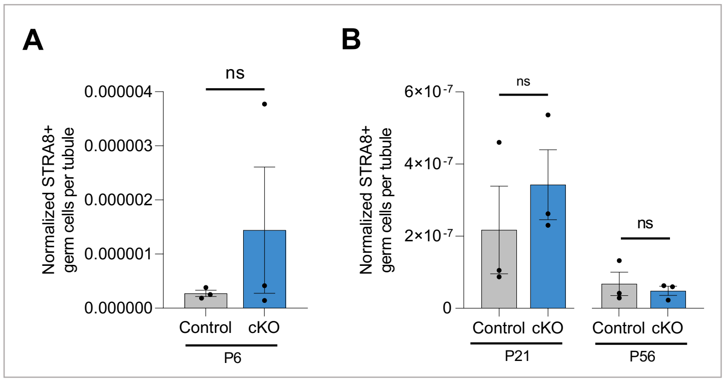

Supplementary Figure 4. Alternative quantification of differentiating spermatogonia with Blimp1-*Cbfb* cKO. (**A**-**B**) Quantification of STRA8+ DDX4+ germ cells per tubule cross-section. Values are normalized to cross-sectional area and presented as mean ± SEM for n = 3 biologically independent animals per genotype and age. Not significant (ns).

Supplementary Figure 5 (connected to Fig. 6)

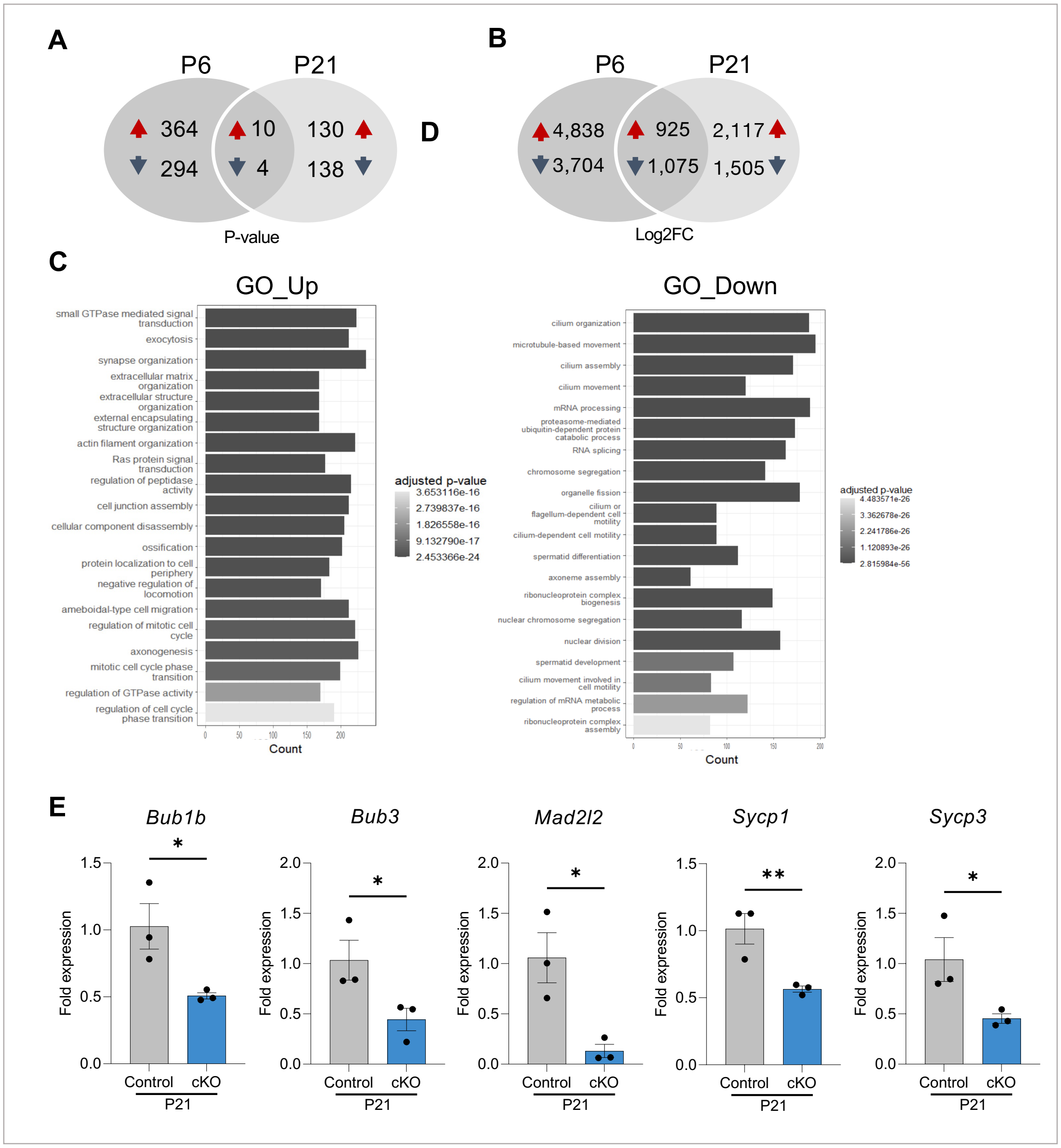

Supplemental Figure 5. Spatial transcriptomics of Blimp1-*Cbfb* cKO. (**A-B**) DEGs with Blimp1-*Cbfb* cKO shared between P6 and P21 undifferentiated spermatogonia (LIN28A+ DDX4+ SYTO13+ cells) based on *p*-value ($p<0.05$; A) or log2FC (>2 log2FC cutoff; B). (**C-D**) Gene ontology of up-regulated (C) and down-regulated (D) DEGs with Blimp1-*Cbfb* cKO based on $p<0.05$ in P21 the differentiating spermatogonia and advanced germ cell populations (LIN28A- DDX4+ SYTO13+ cells). (E) Fold expression of *Bub1b*, *Bub3*, *Mad2l2*, *Sycp1*, and *Sycp3* expression via qPCR between heterozygous control and Blimp1-*Cbfb* cKO testes.
